# Childhood infections, but not early life growth, influence hearing in the Newcastle thousand families birth cohort at age 14 years

**DOI:** 10.1186/1472-6815-13-9

**Published:** 2013-07-29

**Authors:** Fiona Pearson, Kay D Mann, Raphael Nedellec, Adrian Rees, Mark S Pearce

**Affiliations:** 1 Institute of Health & Society, Newcastle University, Newcastle upon Tyne, UK; 2 Ecole Nationale de la Statistique et de l’Analyse de l’Information, Rennes, France; 3 Institute of Neuroscience, Newcastle University, Newcastle upon Tyne, UK

**Keywords:** Childhood hearing, Fetal growth, Risk factors, Aetiology, Epidemiology

## Abstract

**Background:**

While current research priorities include investigations of age-related hearing loss, there are concerns regarding effects on childhood hearing, for example through increased personal headphone use. By utilising historical data, it is possible to assess what factors may have increased hearing problems in children in the past, and this may be used to inform current public health policies to protect children against hearing loss and in turn reduce the long-term burden on individuals and services that may possible evolve. The aim of this study was to investigate which factors in early life significantly impacted on hearing level in childhood using existing data from the Newcastle Thousand Families Study, a 1947 birth cohort.

**Methods:**

Data on early life factors, including growth, socio-economic status and illness, and hearing at age 14 years were collated for a representative subset of individuals from the cohort (n = 147). Factors were assessed using linear regression analysis to identify associations with hearing thresholds.

**Results:**

Males were found to have lower hearing thresholds at 250 Hz, 500 Hz and 1 kHz. Main analyses showed no associations between hearing thresholds and early life growth or socio-economic indicators. An increasing number of ear infections from birth to age 13 years was associated with hearing thresholds at 250Hz (p = 0.04) and 500Hz (p = 0.03), which remained true for females (p = 0.050), but not males (p = 0.213) in sex-specific analysis. Scarlet fever and bronchitis were associated with hearing thresholds at 8 kHz. After adjustment for all significant predictors at each frequency, results remained unchanged.

**Conclusions:**

We found no associations between childhood hearing thresholds and early life growth and socio-economic status. Consistent with other studies, we found associations between childhood infections and hearing thresholds. Current public health strategies aimed at reducing childhood infections may also have a beneficial effect upon childhood hearing.

## Background

Hearing loss is a major public health issue, the magnitude of which is under recognised. The World Health Organisation estimates that there are 275 million people globally with a hearing impairment [1]. Within the UK, there are approximately 10 million individuals with an impaired hearing status, a third of whom are of working age and lower [2]. Hearing loss has a physical, emotional and economic impact upon the individual, as well as an economic impact on society [3-5]. It can lead to high levels of social isolation and an increased risk of mental health problems [5]. While current research priorities include investigations of age-related hearing loss, there are concerns regarding childhood hearing and whether or not it may be affected by early-life factors, infections and modern-day leisure or ‘personal’ noise exposure, for example through listening to heavily amplified music in social settings or to personal music players at high volumes for long periods [6-11]. Childhood hearing loss can lead to lower than average educational attainment and decreased chances of future employment [3,12-14], both of which can have long-term consequences for both the individual and society in general.

Many causes and risk factors for impaired hearing status in childhood have been suggested, for example, predisposing genetic traits, infectious or ototoxic intrauterine exposures (cytomegalovirus, rubella or alcohol), perinatal complications (hypoxia), postnatal infections (otitis media, meningitis, measles), postnatal trauma (head injury, damage to auditory canal) and ototoxic medication use. However, 30-40% of children have hearing loss associated with no known cause or risk factor [12,15-17]. There is also evidence suggesting that a range of factors in early life including adverse socio-economic and familial conditions and early growth play a role in childhood hearing status [18-22].

By utilising historical data from the Newcastle Thousand Families Study birth cohort [23,24], it is possible to assess what factors may have increased hearing problems in children in a population that experienced low childhood exposure to leisure noise in the form of amplified music. The aim of this study was to investigate which factors in early life, including growth, being breast-fed and childhood infections, significantly impacted on hearing thresholds in childhood at age 14 years.

## Methods

### Study participants

The Newcastle Thousand Families Study began as a prospective study of all children born in May and June 1947 to mothers resident in Newcastle upon Tyne, UK (n = 1142) [23,24]. The health, growth and development of the cohort were followed in great detail up to age 15 [23]. Throughout the first years of the children’s lives, all families were visited on a routine (up to every six weeks during infancy and at least quarterly until age five) and an *ad hoc* basis by the study team, which consisted of health visitors and paediatricians [23].

### Measurements from birth to 14 years

Information on early life was recorded prospectively for all study members. Birth weights, recorded by the midwife at the time of the child’s birth, were standardised for gestational age and sex [25]. Duration breast fed (all individuals in this study were breast-fed for at least some time) was defined as the length of time a study member was at least partly breast fed, as recorded by the health visitors. Socioeconomic status at birth (I to V, with I assumed to be the most advantaged and V the least advantaged) was measured by paternal occupational social class at the time of the child’s birth. Housing conditions were assessed by the city’s Public Health Department near the time of the child’s birth, and scored for the presence of overcrowding, lack of hot water, toilets shared between households and dampness or poor repair. Records of childhood infections and illnesses were obtained from formal examinations by a paediatrician at the end of the first, third and fifth years. During the school years, visits were made at least once a year to record height, weight and any health problems up to the age of 15 years in 1962. Additional data from GP and hospital consultations were provided to the study team. Infections from birth to 13 years, and considered in analyses, consisted of measles, mumps, whooping cough, scarlet fever, chicken pox, rubella, tuberculosis and ear infections. Respiratory infection during the first year of life included tonsillitis, bronchitis and pneumonia. Infection events, for all but ear infections, were collated for each individual and grouped into “ever had a specified infection” or “never had a specified infection”. Ear infections were not grouped due to the larger range of numbers of infections seen, so are included as counts.

Hearing thresholds were assessed at age 14 years (1961–62) and measured for 147 study members using pure-tone audiometry at eight frequencies between 250 Hz and 8 kHz and recorded on Amplivox audiogram forms. In line with current British Society of Audiology guidelines, hearing thresholds better than 0 dB HL (i.e. negative values) were given a zero value. An intra individual average (IIA) hearing threshold was calculated as a weighted mean between the ‘best’ and ‘worst’ ear measurement in the ratio 4:1 [26]. Pure tone average (the average of 500 Hz, 1 kHz, 2 kHz and 4 kHz) in both ears was also calculated [27].

Ethical approval for the study was obtained from the appropriate local research ethics committees, the lead of which was the Joint Newcastle Health Authority / University of Newcastle upon Tyne Ethics Committee (ref 94/146), for the analysis of the data and all participants and a parent or legal guardian gave their informed consent at the time of the original data collection.

### Statistical analysis

The representativeness of the participants in this study compared to the remainder of the original cohort was assessed using t, Mann–Whitney and chi-squared tests as appropriate for measures taken during early life. Relationships between IIA hearing thresholds at each frequency and pure tone average and explanatory variables were estimated by linear regression, as were potential interactions between explanatory variables. Initially, univariate linear regression models were investigated for each explanatory variable and significant associations deemed by p < 0.05. Multivariable models were then investigated for all significant associations found, with the final models for each frequency only including significant factors. Additional separate analyses were carried out for males and females. All statistical analyses were done using the statistical software package Stata, version 12 (StataCorp, College Station, TX).

## Results

In the 147 study members that participated in the audiometric examinations, there were 83 males and 64 females with complete data (Table 1). Representativeness of the original cohort was achieved for standardised birth weight (p = 0.687), gestational age (p = 0.148), breast feeding duration (p = 0.583), socioeconomic status at birth (p = 0.604) and sex (p = 0.160). There was, however, a higher percentage with disadvantaged housing conditions in this sample when compared to the remainder of the original cohort.

**Table 1:** Descriptive statistics by sex for all categorical variables

**Variable**		**Male (n)**	**Female (n)**	**Total (n)**
**Sex**		83	64	147
**Social class at birth**	I,II	13	1	14
	III	43	36	79
	IV,V	27	27	54
**Housing conditions at birth**	0	28	24	52
	1	18	19	37
	2 or more	37	21	58
**Duration breast fed**	< 4 weeks	21	16	37
	4 wks-6 mts	40	29	69
	> 6 months	22	19	41
**Measles 0 to 13 years**	No	11	9	20
	Yes	72	55	127
**Mumps 0 to 13 years**	No	59	39	98
	Yes	24	25	49
**Whooping cough 0 to 13 years**	No	25	21	46
	Yes	58	43	101
**Tuberculosis 0 to 13 years**	No	75	55	130
	Yes	8	9	17
**Scarlet fever 0 to 13 years**	No	78	58	136
	Yes	5	6	11
**Chicken Pox 0 to 13 years**	No	29	21	50
	Yes	54	43	97
**Rubella 0 to 13 years**	No	57	45	102
	Yes	26	19	45
**Overcrowding in first year**	No	45	36	81
	Yes	38	28	66
**Tonsillitis in first year**	No	80	63	143
	Yes	3	1	4
**Bronchitis in first year**	No	62	47	109
	Yes	21	17	38
**Pneumonia in first year**	No	78	59	137
	Yes	5	5	10

IIA hearing thresholds ranged from 0 to 47 dB HL across all frequencies, medians and inter-quartile ranges for average cohort hearing at each frequency are outlined in Table 2. The average difference in hearing thresholds between ears for individuals in this cohort was 1.37 dB HL. Males had lower IIA hearing thresholds at frequencies 250 Hz, 500 Hz and 1 kHz than females. Univariate analyses showed no significant associations between hearing thresholds at any frequency and any of the early life growth or socio-economic indicators. The total number of ear infections from birth to age 13 years was associated with hearing thresholds (Table 3) at the lower frequency levels (250 Hz, 500 Hz) which remained true for females (p = 0.050), but not males (p = 0.213) in sex-specific analysis. Using a categorical term for ear infections (0,1,2+) showed no association with hearing thresholds. Scarlet fever and bronchitis had significant associations, all increasing hearing thresholds, but limited to the higher frequencies tested (3 kHz, 4 kHz, 8 kHz) (Table 3). In sex-specific analyses, scarlet fever remained significantly predictive of hearing thresholds, but only in females, (3 kHz p = 0.026; 4 kHZ p = 0.033; 8 kHz p = 0.035), while bronchitis remained predictive for the males only (8 kHz p = 0.005). After adjustment for all significant predictors (i.e. other variables significant for that frequency, visible in Table 3), results remained unchanged. The only significant association with pure tone average was for sex, with males having better hearing than females. No significant interaction terms were found.

**Table 2:** Descriptive statistics by sex for all continuous variables

**Variable**	**Male**			**Female**			**Total**		
**Average intra-individual hearing threshold (dB HL) at**	**n**	**Median**	**IQR**	**n**	**Median**	**IQR**	**n**	**Median**	**IQR**
**250Hz**	83	4	1,7	64	7	1,11	147	6	1,9
**500Hz**	83	5	1,10	64	6	3,11	147	5	1,11
**1 kHz**	83	0	0,1	64	0	0,4	147	0	0,2
**2 kHz**	83	0	0,1	64	0	0,2	147	0	0,1
**3 kHz**	83	1	0,5	64	2	0,7	147	1	0,6
**4 kHz**	83	0	0,1	64	0	0,1	147	0	0,1
**6 kHz**	83	3	0,6	64	3	0,11	147	3	0,7
**8 kHz**	83	1	0,3	64	1	0,7	147	1	0,5
**Gestational age (weeks)**	83	40	40,40	64	40	40,40	147	40	40,40
**Standardised birth weight**	83	−0.37	−1.0, 0.2	64	0	−0.6, 0.8	147	−0.25	−0.8,0.5
**Birth weight (kg)**	83	3.4	3.1,3.7	64	3.37	3.1,3.8	147	3.4	3.1,3.7
**Total number of ear infections, birth to 13 years**	83	0	0,2	64	0	0,1	147	0	0,2

*IQR* Inter-quartile range.

**Table 3:** Adjusted^* ^**regression results: significant associations**

**Variable**	**Frequency (Hz)**	**co-efficient**	**95% CI**	**p-value**
Sex (reference, male)				
	250	2.49	(0.84, 4.15)	0.003
	500	2.01	(0.26, 3.76)	0.024
	1000	1.14	(0.12, 2.17)	0.030
Total number of ear infections, birth to age 13 years				
	250	0.43	(0.01, 0.85)	0.045
	500	0.50	(0.06, 0.94)	0.027
Scarlet fever, birth to age 13 years (reference, No)				
	3000	5.62	(1.56, 9.74)	0.007
	4000	4.20	(0.75, 7.65)	0.017
	8000	4.43	(0.05, 8.82)	0.048
Bronchitis in first year (reference, No)				
	8000	3.09	(0.45, 5.72)	0.022

^*^Results adjusted for all other significant associations at the corresponding frequency (i.e. bronchitis is adjusted for scarlet fever only at 8000 Hz, while ear infections are adjusted for sex only at both 250 and 500 Hz).

### Outlier analysis

Residuals were checked in line with standard checks of linear regression assumptions. One study member had outlying data with high IIA hearing thresholds at all frequencies (range 32–47 dBHL) and a short gestation period (31 weeks). Looking at hearing thresholds separately for each ear at each frequency, the individual had significant unilateral hearing impairment and thus gave a high leverage point in regression diagnostics (shown in leverage versus residual squared plots).

Further to the associations previously outlined when this study member was included, birth weight (standardised for sex and gestational age) was significantly and negatively associated with IIA hearing thresholds at frequencies 250 Hz, 500 Hz, 1 kHz, 3 kHz and 6 kHz. After adjustment for other significant associations standardised birth weight remained negatively associated with hearing thresholds at frequencies 250 Hz, 500 Hz, 1 kHz and 3 kHz, but again was limited to females. Gestational age at birth was associated with hearing thresholds at all frequencies except for 8 kHz. After adjustment for other significant predictors these associations remained, but in sex-specific analyses were limited to females.

## Discussion

Within this birth cohort, males at age 14 years were seen to have lower hearing thresholds than females. Our analyses (excluding an individual with high leverage, outlying data) showed no associations between standardised birth weight and hearing thresholds or between gestational age and hearing thresholds. Further to this, no associations between socio-economic factors and hearing thresholds at age 14 years were found. A significant association between the number of ear infections (age 0–13 years) and hearing thresholds at age 14 years was found; with a greater number of infections associated with higher thresholds. An association was also seen between hearing thresholds and having been infected with scarlet fever and, or, bronchitis. No association was seen between hearing thresholds and having had rubella, measles, mumps, tuberculosis and, or, pneumonia. We also investigated associations between pure tone average data for the cohort, early-life factors and infections, finding no significant associations other than a sex difference.

If a female who had outlying measurements for her IIA hearing thresholds and a relatively short gestation was included in the analyses further associations were seen between birth weight, standardised birth weight and gestation. The decision to exclude this individual was based upon their outlying IIA hearing threshold data and their high leverage in regression diagnostics, since leverage data points can make beta co-efficient estimations inaccurate. Thus, any conclusions drawn about explanatory variables related to hearing thresholds with inclusion of this data could be misleading and should be viewed tentatively. However, this does not necessarily mean that associations found when this individual is included in analyses are invalid.

The main strengths of this study are those of the dataset analysed, in particular the longitudinal nature, breadth of and completeness of data collated within the Newcastle Thousand Families study. Data were prospectively collected on early life experience, including gestational age, parity and socio-economic circumstances at birth, as well as other indicators of early life socio-economic, familial and nutritional status. Hearing thresholds at age 14 years throughout low to high frequencies were also assessed prospectively. This meant potential biases that may be introduced when using recalled information were eliminated. This study may also uniquely have assessed risk factors for non-noise induced hearing loss, as during the era in which these individuals were aged 0 to 14 years, it is likely that exposure to hazardous levels of noise would have been lower than for children today due to the inexistence of personal media devices and, probably for this age and era, much less exposure to heavily amplified music. It is possible that, as children, these individuals would have been exposed to other forms of noise, but as they were all below the legal school leaving age in the UK, it is likely that this assessment took place before any industrial-based exposures would have had an impact. As the study included 8 dependent variables and 19 independent variables, this means that 152 univariate associations were tested. Given the large number of tests done, we cannot rule out that some of the reported associations were significant by chance. For each dependent variable, there was only one final model.

As far as we are aware this is the only cohort study to look at associations with childhood hearing thresholds of all magnitudes across both high and low frequencies within a UK setting. A study based on The National Child Development Study (NCDS), a cohort born in 1958, reported hearing thresholds at 7, 11 and 16 years [21,22], but only looked at associations with sex and social class. The median thresholds were lower in our study at age 14 years than in the NCDS at age 11 [21] and 16 years [22]. At age 16 years, the nearest age to our cohort, there was only a marginal effect of sex in the NCDS, but there was a significant difference between the children of fathers in manual and non-manual occupations with the former having higher thresholds. This difference had been noted at age 11 years, but was larger by age 16. An effect of social class was not evident in our data. Another UK cohort study, assessing risk factors for childhood hearing loss, examined risk factors specifically for mild and high-frequency sensorineural hearing loss [28]. Hearing function amongst The Newcastle Thousand Families study was measured using pure tone audiometry in the early 1960’s, and we are unable to determine whether any hearing loss measured was conductive or sensorineural, or whether it was permanent or temporary. However, as cohort members have hearing loss across all frequencies, it is likely that a mixture of conductive and sensorineural hearing loss is being assessed [29]. This may reflect why associations were seen between thresholds and both scarlet fever and bronchitis.

Consistent with findings from Hall et al’s UK cohort study [28], we found neither birth weight, nor gestation to be associated with childhood hearing thresholds [28]. However, birth weight associations with hearing thresholds in the past have been in relation to sensorineural hearing loss, rather than conductive [19]. Also, consistent with Hall et al’s study [28], we found no association between socio-economic factors and hearing thresholds. However, other studies have found socioeconomic markers to be risk factors for hearing loss [21,22,30] and early growth to be associated with hearing thresholds in later childhood [20,31-33]. No association was seen between hearing thresholds and a number of infectious and congenital diseases previously linked to hearing loss (rubella [34], measles [35], mumps [35], tuberculosis [36] and pneumonia [37]). We were unable to assess whether timing of infections could play a more important role.

The differences seen between our study and others on hearing thresholds may be real or perhaps reflect a lack of power within our study to detect small effects. While significant associations were seen for the non-grouped form of number of ear infections, none were seen using the categorical term. This is likely to reflect the information and variability lost when collapsing the number of infections into just three groups. In addition to the small sample size, this is likely to reflect the difficulties introduced by an average measure when different associations were seen at different frequencies. Younger cohorts living in more economically developed nations now have much lower incidence rates of many of the diseases included in this study than this cohort would have had. This is in part due to vaccinations, but also due to improved living conditions. Hence, it is unclear how the results of the current study, obtained from an historical, pre-vaccinated cohort living in very different conditions to today’s children, would transfer to modern populations. Nevertheless, rates of scarlet fever within the UK are currently at the highest rates for nearly 20 years [38], and diseases such as measles and whooping cough remain endemic in many parts of the world [39,40], with a current epidemic of whooping cough within the UK [41].

## Conclusions

Current public health strategies aimed at reducing childhood infections further may have a beneficial effect upon childhood hearing. Further research is needed in this area to clarify the links between infections, socio-economic status, childhood exposure to recreational noise and fetal growth on later hearing status.

## Competing interests

The authors declare that they have no competing interests.

## Authors’ contributions

MP and AR acquired the data and conceived the idea for this study. RN, KM and FP analysed the data under supervision of MP and AR. FP drafted the paper with critical revisions and final approval from all authors. All authors read and approved the final manuscript.

## Pre-publication history

The pre-publication history for this paper can be accessed here:

http://www.biomedcentral.com/1472-6815/13/9/prepub
